# Two-screw osteosynthesis is biomechanically superior to single-screw osteosynthesis for type II odontoid fractures

**DOI:** 10.1038/s41598-024-69905-3

**Published:** 2024-08-15

**Authors:** Paul Jonathan Roch, Elisa Salge, Miguel Angel Bravo Cabrera, Friederike Sophie Klockner, Marc-Pascal Meier, Katharina Jäckle, Wolfgang Lehmann, Lukas Weiser

**Affiliations:** Department of Trauma Surgery, Orthopaedics and Plastic Surgery, University of Göttingen, Robert-Koch-Str. 40, 37075 Göttingen, Germany

**Keywords:** Odontoid fracture, Osteosynthesis odontoid, Anterior approach, One screw, Two screws, Biomechanical, Medical research, Diseases, Trauma

## Abstract

The data on the use of a one- or two-screw technique (1S, 2S) for ventral osteosynthesis of type II dens fractures are contradictory. The aim was to design an apparatus to mimic the physiological conditions and test stability with 1S, 2S, and a headless compression screw (HCS) for osteosynthesis of artificially created type II odontoid fractures. The apparatus was mounted on a Zwick materials testing machine. A total of 18 C1–2 specimens were stratified into three groups (1S, 2S, HCS). Odontoid fractures were artificially created, and osteosynthesis was performed. Each specimen was tested at loads increasing from 1 to 40 N. Screw loosening was observed visually, by fatigue data, and by a camera tracking system. Analysis of the Zwick data and the camera data revealed a significant higher stability after 2S compared to 1S and HCS treatment (Zwick data: p = 0.021, camera data: p < 0.001), while visible screw loosening showed a superiority of the 2S only over HCS (p = 0.038). The developed apparatus allowed the dynamic study of the atlantoaxial joint with a high approximation to physiological conditions. The results demonstrated superiority of the 2S over the 1S and HCS in biomechanical stability in the treatment of type II odontoid fractures.

## Introduction

Current research provides conflicting results as to whether the one- or two-screw technique (1S, 2S) provides superior stability and lower nonunion rates for the treatment of type II odontoid fractures. From a clinical perspective, it has been observed that retrospective studies using dynamic radiographs have shown no significant differences in stability between 1 and 2S^1^. However, some studies have suggested that there may be increased stability with 2S^2^. According to the AO Surgical Reference, a 2S will improve rotational stability, but in younger patients with robust bone quality, a 1S may be sufficient^3^. However, a recent meta-analysis showed a higher screw removal rate with the 2S^4^. From a biomechanical perspective, some studies that have focused solely on C2 have suggested that 2S may be superior to 1S^5^, while others have found no significant differences between the two techniques^6,7^.

Although rotation and flexion/extension are crucial atlantoaxial joint movements, they frequently involve combined movements. Puttlitz et al.^8^ designed a finite element model indicating that a combination of lateral shear and axial rotation causes type II odontoid fractures. Moreover, single movements may not apply sufficient strain on screw osteosynthesis for type II odontoid fractures, necessitating more complex movements in C1–2 specimens.

The purpose of this study was to develop and establish an apparatus for a Zwick materials testing machine (145.660 Z020/TND; Ulm, Germany; Zwick) to simulate the physiological and complex movements of the embedded C1–2 specimens, taking into account all three dimensions of motion in a dynamic movement. The study then compared the stability of the 1S and 2S for artificially induced odontoid fractures, with a third group testing thicker headless compression screws (HCS).

## Materials and methods

### Specimens

The research was approved by the Institutional Review Board of the University of Göttingen under the Ethics Committee Göttingen with approval number 28/08/23. All experiments were performed in accordance with relevant guidelines and regulations. Informed consent was deemed unnecessary because the donors had previously bequeathed their bodies to the Center of Anatomy of the University of Göttingen, which assumed their capacity to give unrestricted consent. A total of 18 human functional spinal units (FSUs) C1–3 were obtained from these donors.

Computed tomography (CT) scans were performed to exclude specimens with signs of osteochondrosis, spondylarthrosis, or injury and to measure bone mineral density. Soft and muscular tissues, vessels, and neural structures were then removed from the specimens, leaving the ligaments and capsules intact. These specimens were immediately frozen at − 20° C and stored until the start of the experiment.

The FSUs were stratified into three groups according to age and sex. After thawing, an artificial Anderson d'Alonzo II type, Eysel and Roosen type A odontoid fracture was created with an 1 mm thick oscillating saw under fluoroscopic guidance from anterior, as described in the literature^5–7^. Osteosynthesis was performed by an experienced spine surgeon who was not involved in the subsequent phases of the study as follows: Group I (1S) received a 4.0 mm cannulated screw (DePuy Synthes, PA, USA), Group II (2S) was treated with 2 4.0 mm cannulated screws (DePuy Synthes), and Group III (HCS) was treated with a 5.0 mm Fixos screw (Stryker, MI, USA). Screw length selection was based on CT scan results. All screws were placed bicortically.

C2 and C3 were fixed with standard screws to allow adequate mobility between C1 and C2 when positioned within brackets. The FSUs C1-3 were then secured within brackets using a cold polymerization agent (Weitur-Press Standard, Weithas, Lütjenburg, Germany). During embedding, the upper endplate of C2 was horizontally aligned with the brackets. Sagittal alignment was obtained by drawing a virtual line through a point at the center of the spinous process and the center of the C2 vertebra. Coronal alignment was achieved by placing the posterior wall of the vertebra in the center of the bracket. Throughout the experiment, the specimens were periodically moistened to prevent desiccation.

### Testing apparatus

**Figure 1:**
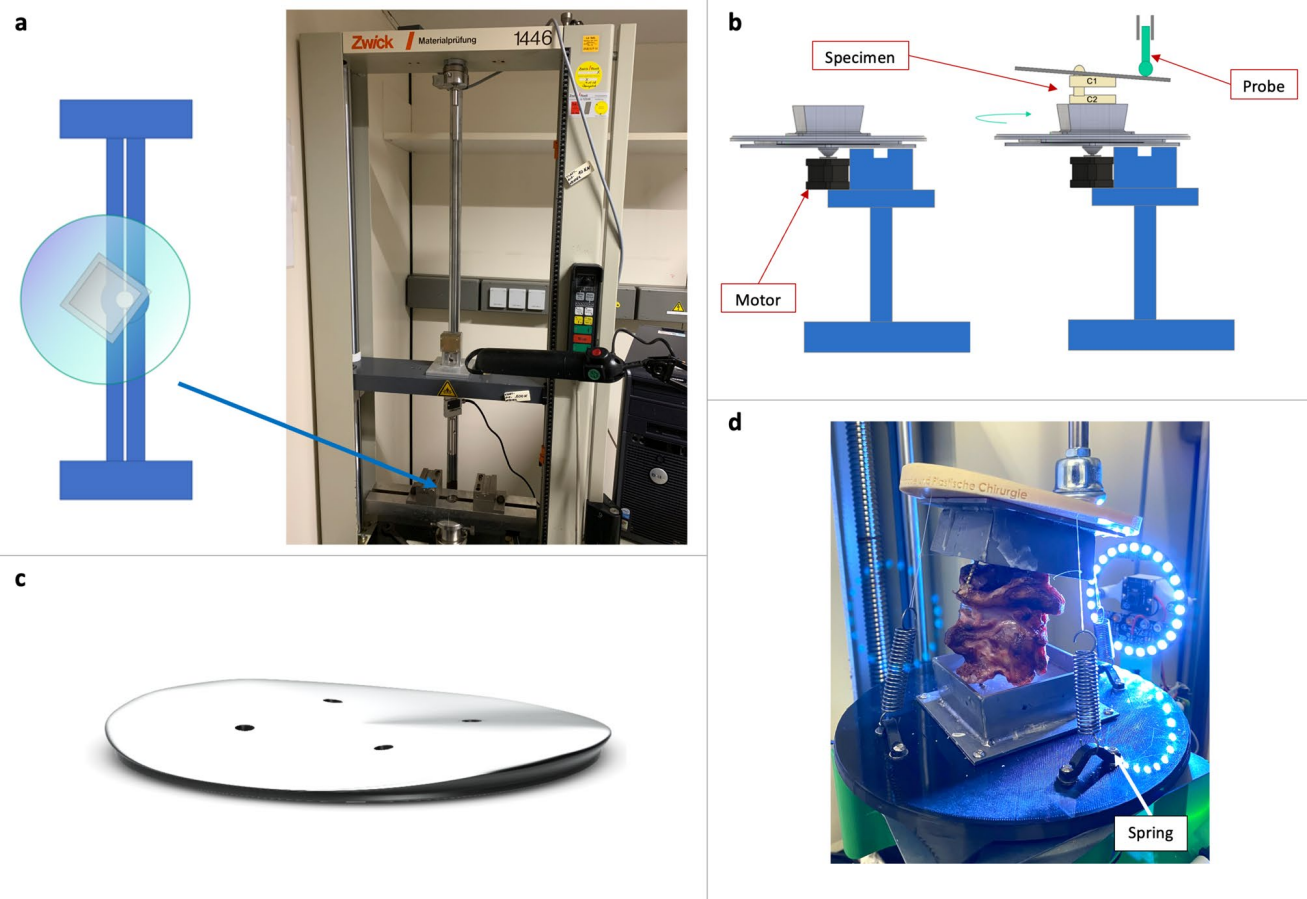
Schematic model of the apparatus. (**a**) The blue design schematically represents the lower bar of the Zwick. (**b**) The main part of the apparatus is a motor-driven turntable that is placed on this lower bar. The specimens were embedded in special brackets on the turntable and on the top plate, which was placed on C1. The Zwick’s probe exerted forces on the top plate. (**c**) The top plate was deliberately designed with a symmetrical curvature. Both the anterior and posterior sections were accurately elevated by 5 mm, resulting in a 4.8° variance compared to the lateral aspects on a 12 cm diameter of the top plate. This design choice was made to accommodate the greater range of flexion and extension movements as opposed to lateral flexion^19,20^. **d**) Photograph of the apparatus with a specimen embedded during the experimental run.

The critical part of the apparatus was a motorized rotating plate on which the specimens could be mounted in brackets. The rotations could be changed. Another plate was placed horizontally on top of the specimen. Here, the Zwick’s probe applied forces for lateral bending and flexion/extension movements. In total, a dynamic motion was induced with all 3 dimensions of motion and additional shear forces (Fig. 1).

**Figure 2:**
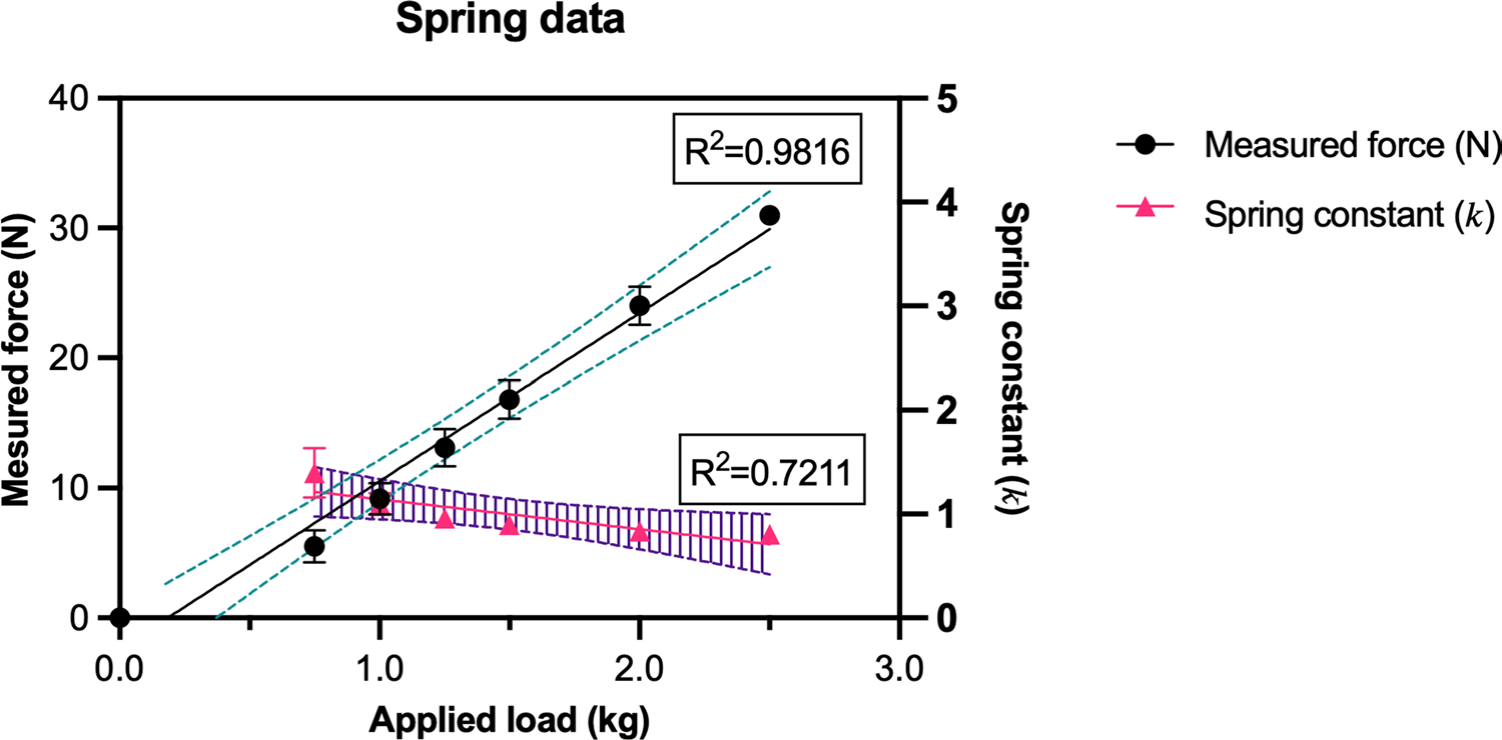
Spring characteristics. Spring characteristics are shown, when load was applied on the springs (LTZ 2 maximum load 20 N, readability: 0.4 N / 0.04 kg G&G GmbH, Kaarst, Germany). The linear regression analysis showed an excellent goodness of fit for the measured force (R^2^ = 0.9816). The goodness of fit of the linear regression for the spring constant (*k*) was R^2^ = 0.7211. However, the maximum load for the springs was specified to be 20 N. Therefore, calculating the linear regression only up to 15 N resulted in a goodness of fit of R^2^ = 0.9035.

For simulation of head weights, four springs were placed between the lower and top plates. The average head weight was approximated to be 5 kg^9^. The springs were adjusted to provide a balanced preload of 50 N at rest, with each spring set at 12.5 N. As the top plate was pressed down laterally with the Zwick’s probe, the opposite spring was extended, applying additional loads (Fig. 2). The springs were changed after each test run.

### Experimental tests

**Figure 3:**
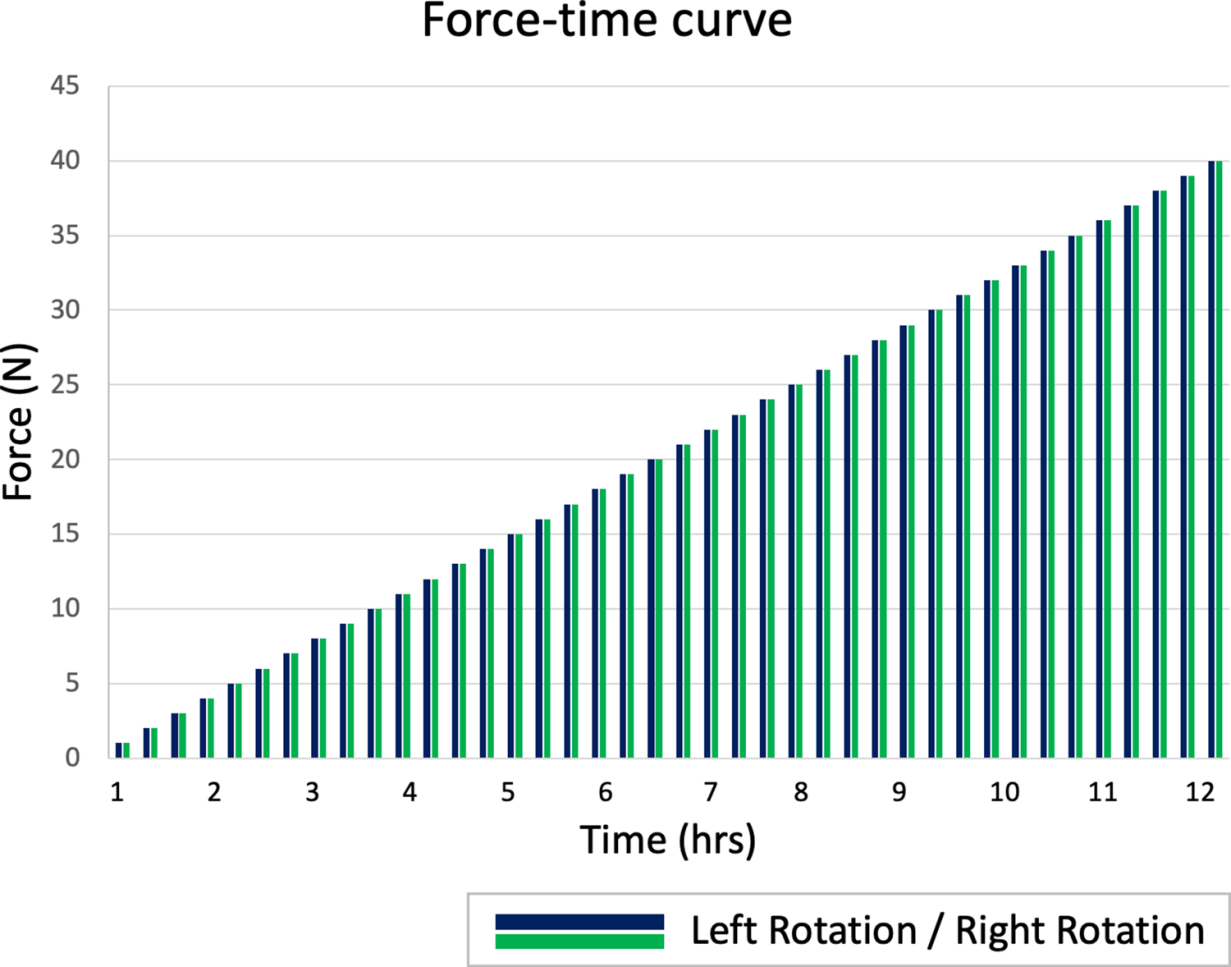
Approximated application of force over time. The Zwick’s probe started with the application of a load of 1 N. The first direction of rotation of the turntable was randomized^10^. The direction was changed after the specific load was applied 100 times. The entire load application lasted about 12 h.

The experiments were conducted as fatigue tests. The first direction of rotation of the turntable was randomized^10^. The Zwick’s probe started 3 mm above the elevated anterior part of the top plate. Then, when the experiment started, it approached this top plate at a speed of 500 mm/min. When contact applied a load of 1 N, the Zwick immediately changed the direction of the probe and retracted it again at a speed of 500 mm/min to the start location, which was 3 mm above the highest part of the symmetrically curved top plate. This cycle was repeated 100 times. The turntable rotated 3.33 times per minute. Next, the rotation of the turntable was changed, and the entire procedure was repeated. The force was then increased in increments of 1 N to 40 N (Fig. 3). When there was no visible evidence of screw loosening, the force was increased to 50 N.

### Optical tracking system

**Figure 4:**
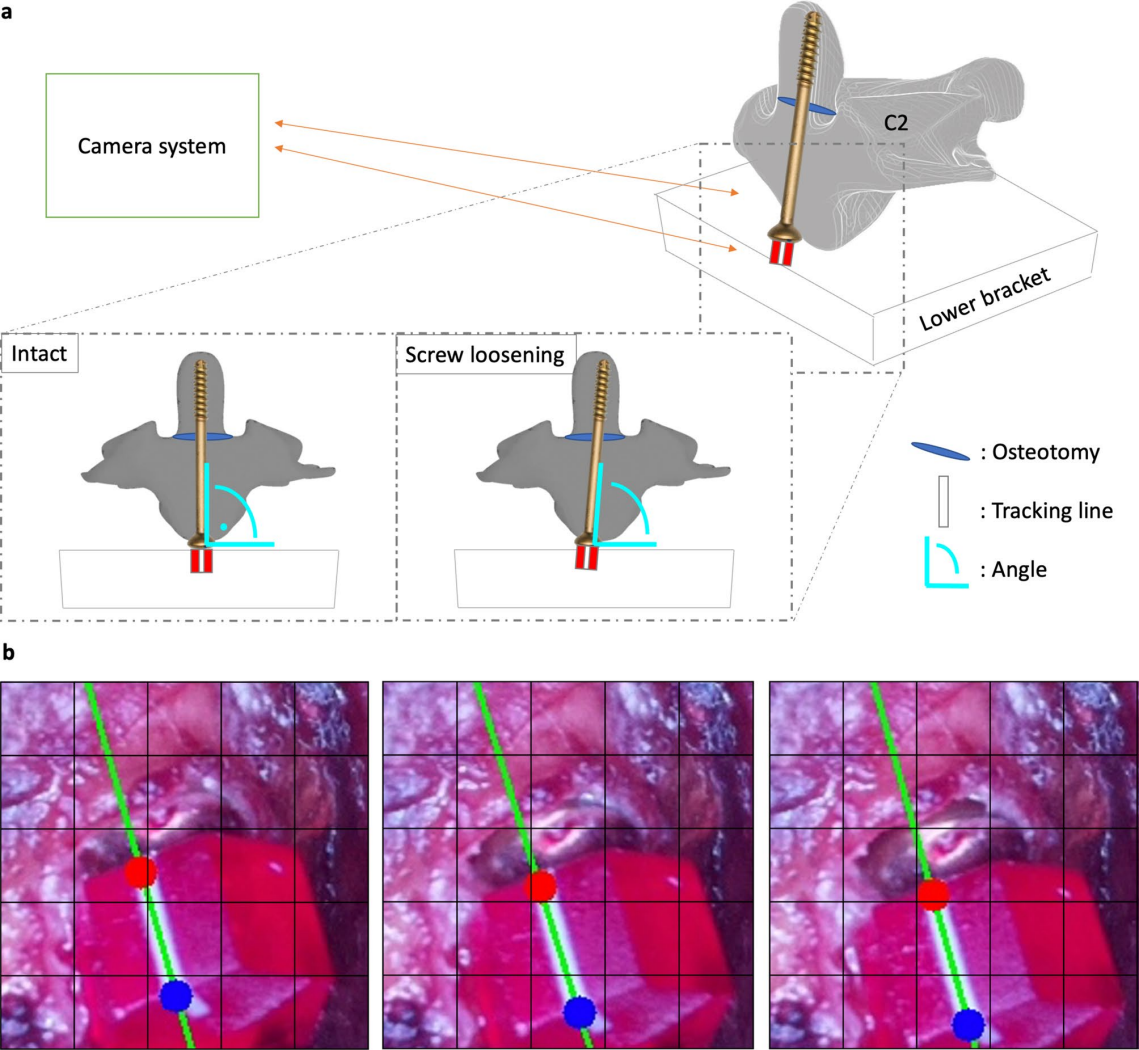
Camera tracking system. (**a**) The camera tracking system used a red cube with a white line attached to the top of the screw. A photograph was taken at the same location for each rotation of the turntable. (**b**) An algorithm provided the coordinates of the two endpoints (red, blue) of the white tracking line. Changes in the location of each point were used to determine the point of screw loosening.

The duration until the point of fatigue was not foreseeable, so an optical tracking system was applied to the construction (Fig. 4A). Therefore, a cube with a tracking line of only a few milligrams was attached to the top of the screw. Owing to its light weight, it had no relevant impact on the experiment. The camera system monitored the position of the tracking line in each circle of the turntable at a fixed position. Whenever the screw rotated or lost hold, the spatial arrangement of the tracking line changed (Fig. 4B).

### Image processing algorithm

The image analysis method is based on a self-developed algorithm written in PyCharm 2023.1 (JetBrains, Prague, Czech Republic).

A colored marker attached to the screw served as the focal point of the analysis, facilitating precise evaluation of the screw’s movement. Essential parameters, such as the screw identifier and the image directory, were requested from the user, thus establishing a basic framework for the subsequent analysis. The user marked the position of the screw in the first image of the series. Thus, the algorithm identified key reference attributes, such as the marker’s location to establish the region of interest, and its color characteristics. This initialization process strategically positions the algorithm to perform automatic marker identification in subsequent images, calibrated by the initial user-defined references.

In the preprocessing phase, each image in the series underwent a normalization process with a color transfer technique using the first image in the series as a reference. Through this technique, the algorithm optimized the brightness and contrast of each image by calibrating these to the color characteristics of the initial image. This ensured that the visual characteristics would be as consistent as possible throughout the analysis.

The algorithm could now automatically detect the marker. Using the color thresholds obtained from the initialization phase, a binary mask was generated. This mask was refined using morphological operations such as erosion and dilation to increase the accuracy of the subsequent contour detection process.

Following contour detection, the algorithm performed a marker evaluation process. A line was fitted to the marker contour considered to represent the marker, which facilitated the calculation of the marker’s orientation and position coordinates. The orientation, or angle, was calculated relative to a predefined axis.

A real-time visualization capability was added to the algorithm, allowing dynamic visual inspection of key analysis steps such as the detection of markers and their fitted lines. This visualization enhanced the transparency and verifiability of the analytical process and fostered an environment in which the experimenter could monitor the analysis at all times.

Finally, the algorithm created a comprehensive dataset that encapsulated the nuanced attributes of the marker’s position and orientation. This dataset was structured in a JSON format, cultivating a repository primed for subsequent in-depth analysis and interpretation of the results.

### Statistics

Statistical analyses were performed using GraphPad Prism 9.5.1 (GraphPad Software, San Diego, CA, USA). Analysis of variance (ANOVA) was used because the data were parametric. Differences between groups were analyzed using Tukey’s post hoc test (p < 0.05). Linear regression analysis was performed to evaluate the effect of mineral density on screw loosening. Data are presented as means and standard deviations.

## Results

### Specimen characteristics

Specimens consisted of 4 females and 14 males with a mean age of 81.28 ± 8.54 years. There was no significant difference in age between the sexes (p = 0.844). Bone mineral density was measured at the apex, middle, and base of the odontoid. The apex showed significantly higher bone mineral density compared to the middle and the base in all three treatment groups (p < 0.001). There was no significant difference in mean bone mineral density between the three groups: Bone mineral density was 113.5 ± 14.5 mg/cm^3^ in group 1S, 162.1 ± 16.4 mg/cm^3^ in group 2S, and 115.5 ± 11.0 mg/cm^3^ in group HCS (p = 0.563). There were no differences in bone mineral density between the sexes of the specimens (p = 0.237).

### Screw loosening

**Figure 5:**
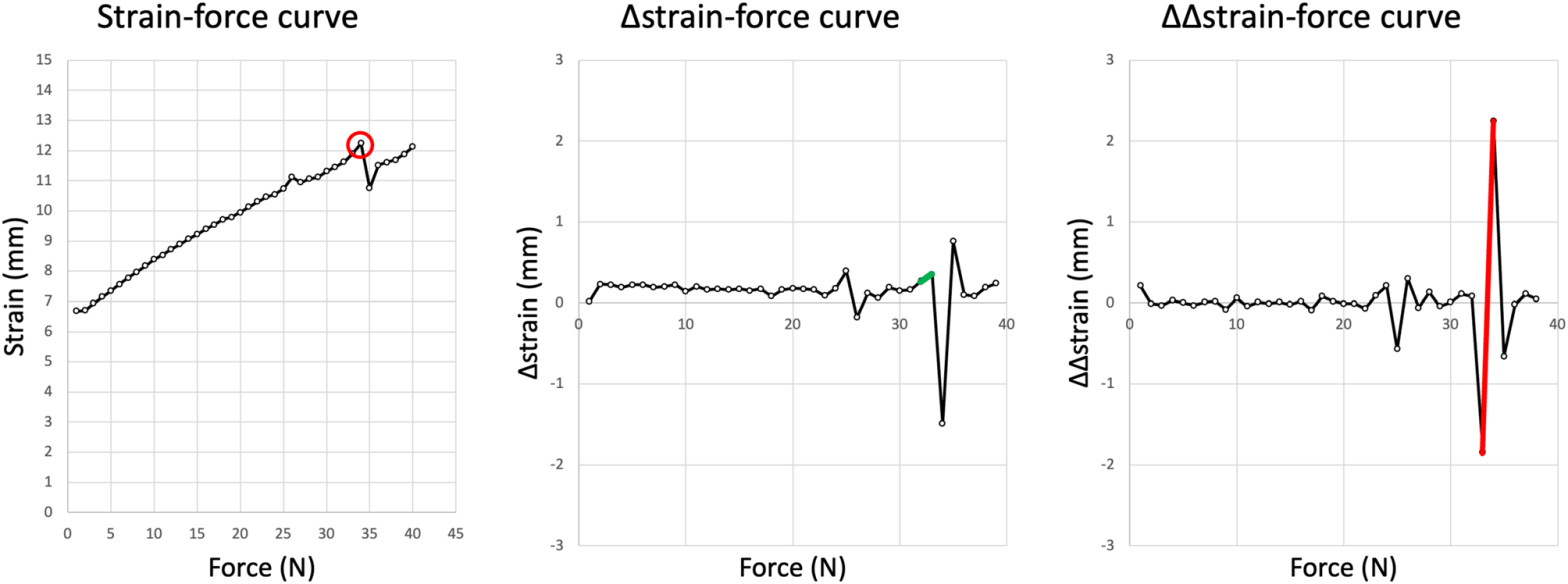
Determination of screw loosening using Zwick data. Example of determining screw loosening from a single specimen. In principle, the optimum strain-force curve was expected to be curved with a steady asymptotic decrease in strain with increasing force. Thus, Δstrain was expected to become smaller with increasing force, and ΔΔstrain was expected to be always negative. In the case of screw loosening, strain would increase, as would Δstrain, and ΔΔstrain would become positive. In the case of screw loosening, the initial loosening was followed by a decrease in strain, most likely due to a jammed screw in the bone. The strain-force curve shows two peaks reflecting a sudden increase and decrease in strain, which were considered potential points of screw loosening. To determine the point of screw loosening mathematically, Δstrain was plotted against force, showing when the change in strain was about to become greater or smaller. As described above, an increase in strain would also result in a positive Δstrain (green line). To determine a significant increase in Δstrain and thus the point of screw loosening, the first positive 50% increase above the mean of the ΔΔstrain values was assumed (the first three test runs [1–3 N] were regarded as a settling phase and calculated points of screw loosening were not counted, but only from > 3 N). In the case shown, this means a positive ΔΔstrain above 50% of the mean at 34 N (red line). This marks the second peak of increased strain in the strain-force curve (red circle).

**Figure 6:**
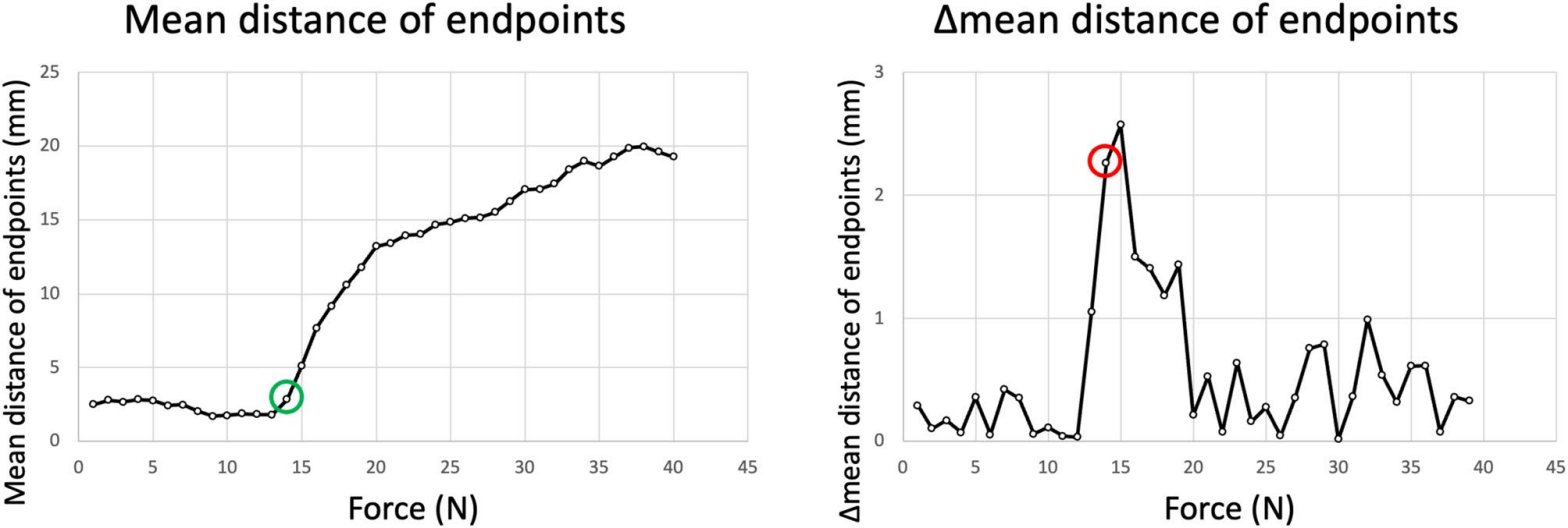
Determination of screw loosening using camera system data. For the camera tracking analysis, the algorithm described above provided the coordinates of the two endpoints (red, blue) of the white tracking line. Using the coordinates of the two endpoints, the distance of the respective coordinate to the reference point of the first image was calculated for each image. The average was then calculated for each force and direction setting (e.g., 1 N, right rotation). This resulted in a total of 80 mean distance values (1–40 N, left/right rotation). Calculations were then performed for the mean of both directions of rotation separately. According to the analysis of the Zwick data, an initial settling (1–3 N) was assumed. Subsequently, under optimal conditions, the calculated mean value should remain the same, and the differences in the mean values between the force levels should be approximately 0. The point of screw loosening was defined when the difference was three times the median of the differences. The median was chosen instead of the mean in order to exclude large distance differences in the case of extreme screw loosening and to obtain a more accurate value to represent the average distance differences before screw loosening. In the case shown, a Δmean three times the median was reached at a force of 14 N (red circle). This marks the beginning of the increase (green circle) and was determined to be screw loosening.

Three methods were used to detect screw loosening: Visible screw loosening detected by the experimenter, fatigue data from the Zwick, and data from the camera tracking system. The methods for the determination of the point of screw loosening from the Zwick’s fatigue data and camera tracking data are elaborated in Figs. 5 and 6.

**Figure 7:**
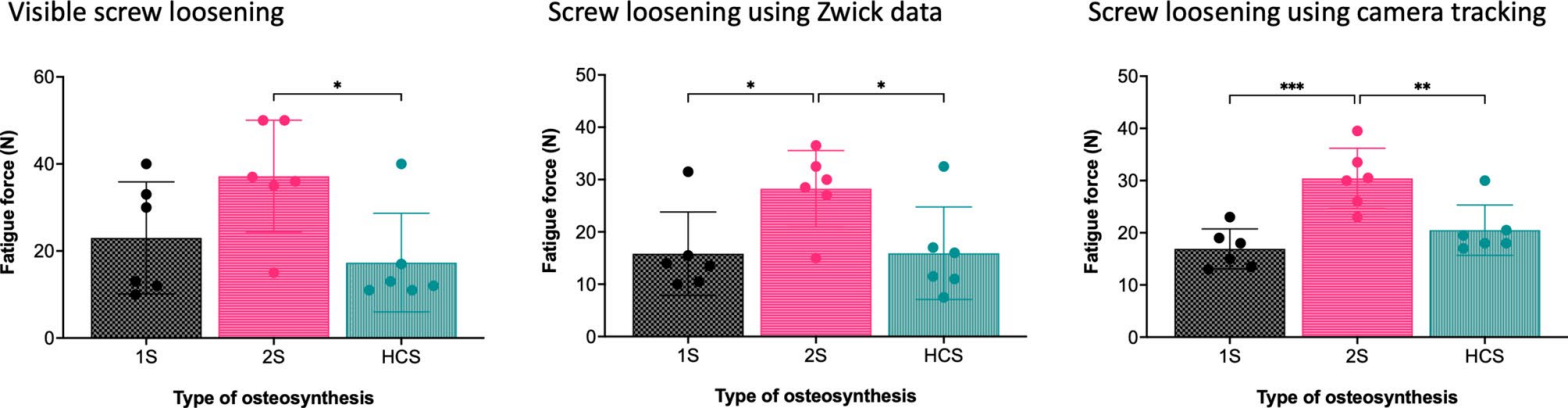
Screw loosening based on observation, Zwick data and camera tracking. Visible screw loosening indicated significantly higher stability of 2S compared to HCS, while both Zwick data and camera tracking showed significantly higher stability after 2S compared to 1S and HCS. The earliest loosened screw was selected for the camera tracking analysis. 1S: one-screw osteosynthesis, 2S: two-screw osteosynthesis, HCS: headless compression screw osteosynthesis. *p < 0.05, **p < 0.0021, ***p < 0.0002.

The visual method showed a significant higher stability of the 2S compared to the HCS. The Zwick data and the data from the camera tracking system indicated a significantly higher stability of the 2S compared to the 1S or HCS (Fig. 7, Table 1). No relevant effect of turntable rotation direction on screw loosening was observed (Table 2). Linear regression analysis showed no effect of bone mineral density on screw loosening using either camera data (p = 0.331) or Zwick data (p = 0.414).

**Table 1:** Screw loosening based on observation, Zwick data, and camera tracking.

	1S		2S		HCS		p
Visible loosening (N; median ± SD)	23.0 ± 12.9		37.2 ± 12.9	^HCS^	17.3 ± 11.3	^2S^	**0.038**
Zwick data (N; median ± SD)	15.1 ± 8.4	^2S^	28.8 ± 7.5	^1S, HCS^	16.6 ± 8.4	^2S^	**0.021**
Camera data (N; median ± SD)							
2S, loosened screw:							
Earliest screw (either screw A or B)	16.9 ± 3.8	^2S^	30.4 ± 5.8	^1S, HCS^	20.5 ± 4.8	^2S^	** < 0.001**
Screw A	16.9 ± 3.8	^2S^	35.3 ± 11.1	^1S, HCS^	20.5 ± 4.8	^2S^	**0.001**
Screw B	16.9 ± 3.8	^2S^	33.7 ± 4.8	^1S, HCS^	20.5 ± 4.8	^2S^	** < 0.001**

Zwick data and camera tracking indicated significantly higher stability after 2S compared to 1S and HCS. In visible screw loosening, significantly higher stability was detected for 2S only compared to HCS. During the right rotation, a significantly higher stability of 2S compared only to 1S was observed, whereas in all other rotation tests, a significantly higher stability of 2S compared to both 1S and HCS was noted. 1S: one-screw osteosynthesis, 2S: two-screw osteosynthesis, HCS: headless compression screw osteosynthesis. ^1S^: significant versus 1S, ^2S^: significant versus 2S, ^HCS^: significant versus HCS.

**Table 2:** Influence of rotational direction on screw loosening.

	Left rotation	Right rotation	p
Zwick data (N; median ± SD)			
1S	12.8 ± 3.4	17.3 ± 13.8	0.455
2S	25.3 ± 7.2	32.2 ± 10.1	0.206
HCS	15.5 ± 9.1	17.7 ± 8.4	0.677
Camera data (N; median ± SD)			
1S	16.0 ± 3.2	17.8 ± 4.7	0.447
2S (screw A)	35.8 ± 13.8	34.8 ± 10.7	0.891
2S (screw B)	29.5 ± 1.8	37.8 ± 8.8	**0.046**
HCS	20.2 ± 3.2	21.6 ± 9.9	0.744

During left rotation and after 2S, screw B was loosened significantly earlier than during right rotation. Otherwise, there were no significant differences between left and right rotation. 1S: one-screw osteosynthesis, 2S: two-screw osteosynthesis, HCS: headless compression screw osteosynthesis.

## Discussion

This study involved the development of an apparatus that allows the atlantoaxial joint to be examined as closely as possible to physiological conditions. Subsequent investigations showed that the 2S is superior to both the 1S and the HCS in terms of biomechanical stability.

The development of a new apparatus to study the atlantoaxial joint seemed necessary because previous biomechanical experiments either examined the odontoid in isolation^5,6^ or, in the case of C1–2 specimens^7^, did not generate a load such as the described combination of lateral shear and axial rotation^8^, which is essentially responsible for causing type II odontoid fractures and therefore also appears to be essential for testing the stability of an osteosynthesis. While the biomechanical studies listed^5–7^ did not reveal any significant difference between the 1S and the 2S, the current study was able to find a clear superiority of the 2S above the 1S and HCS.

A striking feature of the study are the three independent examinations used to investigate stability. While visible screw loosening was only recorded by the observer during the experiment and is therefore only a rough estimate of screw loosening, additional highly sensitive methods of testing stability were established by evaluating Zwick and camera data.

From an intrinsic biomechanical understanding of traumatology, the proof of a higher stability of the 2S compared to the 1S seemed overdue. Not only do two screws alone seem to hold more than one, but the odontoid is, after all, a kind of ball-and-socket joint (where the posterior part is stabilized by ligaments). Similarly to a dynamic hip screw, where an antirotational screw leads to significantly more stability^11^, the 1S lacks anti-rotational forces, which is overcome by the 2S. This is probably where the difference between the current study and the previous ones can be found. For example, in isolated studies of C2^5,6^, no rotational forces were generated on the odontoid. However, the articulation between C1 and C2 via the disc and facet joints results in combined forces^12^ that are likely to be different from single loading tests on the odontoid. Graziano, et al.^7^ examined C1–2 specimens with torsion tests that were conducted at a speed of 1 rotation per second, reaching a rotation angle of 10°. However, it remained with an isolated slow rotational movement, whereas in the present study a combination of lateral shear and axial rotation forces was generated by dynamically applying force to the rotating FSU C1–2 through the Zwick. Since this combination of movements triggers the essential load on the odontoid^8^, the superiority of the 2S over the 1S could be demonstrated. In this context, the study also investigated the influence of the direction of rotation on screw loosening, but it was unable to observe any significant difference here. It can be assumed that the use of FSU C1–2 resulted in a combination of lateral shear and axial rotational forces only at the beginning of a force application, while a continuous rotational movement on the odontoid naturally did not occur.

As mentioned, the study did not observe any significant influence of the direction of rotation on screw loosening. This could be attributed to the use of compression in osteosynthesis. Consequently, the fragments might have been securely fixed together, minimizing any detectable effects of rotation.

Thicker diameter HCSs have also been tested. A correctly selected pedicle screw diameter is essential for stability^13^. Viezens, et al.^14^ suggested that the larger the screw diameter in relation to the pedicle circumference, the greater the potential fatigue load on the pedicle screw. This appears to be due to the hold of the screw on the cortical bone^15^. A similar condition was assumed for osteosynthesis of odontoid fractures. However, the HCS showed significantly less stability than the 2S. In addition to the lack of antirotational moment present in the 2S, the thread at the base of the HCS used was relatively wide, which required a wide cortical opening at the base of the C2 vertebra and may have resulted in reduced fixation.

The osteotomy was performed using a 1 mm thick oscillating saw under fluoroscopic guidance from the anterior to simulate an artificial Anderson-D'Alonzo II, Eysel, and Roosen type A odontoid fracture, as previously described in the literature^5–7^. Two primary factors may have contributed to the observed outcomes, thereby representing a potential limitation of the study. (1) Despite the use of a 1 mm thick saw, the sawing process itself results in bone loss, which in turn affects the positioning of fragments following osteosynthesis. (2) The osteotomy performed with a saw creates a flat surface, which differs from that of a traumatically occurred odontoid fracture. Consequently, the transferability of the results to natural conditions must also be approached with caution in light of these considerations.

Despite the biomechanical results, the clinical question will arise as to the extent to which a 2S can be performed regularly. Even if anterior screw osteosynthesis only requires a small and simple approach^16,17^, nonunion, dysphagia, esophageal and retropharyngeal injuries, and wound hematomas remain relevant complications^18^. Performing a 2S will make the operation significantly more difficult and increase the risk of complications. In particular, the second screw coming from the opposite side is a challenge that places stress on the soft tissues. The use of intelligent implant systems or navigation could be helpful.

## Data Availability

The datasets used and/or analysed during the current study available from the corresponding author on reasonable request.

## Abbreviations

1S: One-screw technique
2S: Two-screw technique
CT: Computed tomography
FSU: Functional spinal unit
HCS: Headless compression screw
Zwick: Zwick materials testing machine (145.660 Z020/TND; Ulm, Germany)
